# Oxidative Stress and Therapeutic Development in Lung Diseases

**DOI:** 10.4172/2161-105X.1000194

**Published:** 2014-07-15

**Authors:** Leah Villegas, Timothy Stidham, Eva Nozik-Grayck

**Affiliations:** 1; 2

**Keywords:** Oxidative stress, Lung disease, Reactive oxygen species, Nitric oxide, Antioxidant

## Abstract

Oxidative stress has many implications in the pathogenesis of lung diseases. In this review, we provide an overview of Reactive Oxygen Species (ROS) and nitrogen (RNS) species and antioxidants, how they relate to normal physiological function and the pathophysiology of different lung diseases, and therapeutic strategies. The production of ROS/RNS from endogenous and exogenous sources is first discussed, followed by antioxidant systems that restore oxidative balance and cellular homeostasis. The contribution of oxidant/antioxidant imbalance in lung disease pathogenesis is also discussed. An overview of therapeutic strategies is provided, such as augmenting NO bioactivity, blocking the production of ROS/RNS and replacement of deficient antioxidants. The limitations of current strategies and failures of clinical trials are then addressed, followed by discussion of novel experimental approaches for the development of improved antioxidant therapies.

## Introduction

Oxidant/antioxidant imbalance has been implicated in the pathogenesis of diseases affecting every organ system, including the lung and pulmonary vasculature. The field has significantly evolved from the early investigations that defined the source of excess production of Reactive Oxygen Species (ROS), identified the antioxidant systems, both enzymatic and non-enzymatic, and established that oxidative stress damages cell structures. These early studies were followed by the discovery of nitric oxide (NO^•^) as a biologic signaling molecule, and the emerging field of redox biology, the study of reactive oxygen and nitrogen species as signaling molecules through specific, regulated and targeted modifications. Numerous clinical trials have tested different strategies to protect against oxidative stress or restore physiologic NO activity in lung and pulmonary vascular diseases, though the results have overall been disappointing. This review article will highlight the major oxidant and antioxidant systems in the lung; provide a framework to understand redox-regulated signaling; review the clinical trials aimed to restore NO bioactivity, scavenge ROS or replete deficient antioxidants in a range of lung diseases; speculate on the reason for the overall insufficient clinical responses; and provide an overview of new therapeutic strategies currently under investigation designed to overcome the limitations with current therapies.

## Overview of ROS/Antioxidants in the Lung

### ROS/RNS production from endogenous sources and their role in lung diseases

Endogenous oxidant-antioxidant systems have an important role in lung diseases. Reactive radical species are ubiquitous in nature, produced from endogenous and exogenous sources. Cellular organelles such as mitochondria and peroxisomes are major sources of reactive oxygen (ROS) and nitrogen species (RNS) [1,2]. In the mitochondrial electron transport chain, unpaired electrons are generated by oxidative phosphorylation, which reduces molecular oxygen, leading to the production of superoxide anion (O_2_^•−^). Superoxide is rapidly reduced to hydrogen peroxide (H_2_O_2_). Peroxisomes are cell organelles that contain oxidases and catalases. These enzymes play a key role in normal metabolic pathways that contribute to the catalysis of ROS and RNS byproducts, implicating peroxisomes as a major source of oxidative stress. Some of the major enzymatic sources of ROS and RNS include flavoproteins that produce H_2_O_2_, and xanthine oxidase and the nitric oxide synthases that produce O_2_^•−^ and NO^•^ [3–5]. A number of other important cellular enzymes such as Nicotinamide Adenine Dinucleotide Phosphate (NADPH) oxidase, lipooxygenases, uncoupled endothelial nitric oxide synthase (eNOS), and cytochrome P450, contribute to the production of ROS/RNS that play a role in lung diseases [6–9]. Non-enzymatic production of reactive species also occurs through metal-catalyzed oxidation such as the Fenton reaction (Fe^2+^ + H_2_O_2_ → Fe^3+^ + OH^−^ + OH^•^) or thermodynamic reactions of NO^•^ with O_2_^•−^ to form peroxynitrite (ONOO^−^) [10,11].

### ROS/RNS production from exogenous sources and their role in lung diseases

Production of reactive species from exogenous sources such as environmental toxins and diet promote the onset of lung diseases. Classical examples of lung injury caused by environmental toxins include exposure to paraquat (a commonly used herbicide) and chronic ethanol consumption. Paraquat poisoning has been shown to induce oxidative stress and increased expression of cystine/glutamate transporter, Nrf-2 regulated mitochrondrial dysfunction, and inflammation in the lung [12–14]. While dietary phenols (i.e. resveratrol) have shown to inhibit paraquat-induced oxidative stress [15], phenols (curcumin and resveratrol) can also regulate oxidative stress and inflammation by activation of nuclear factor kappa-light-chain-enhancer of activated B cells (NF-κB) and activator protein 1 (AP-1) [16]. Chronic ethanol consumption is associated with increased incidence of Acute Respiratory Syndrome (ARDS), where one proposed mechanism is the up-regulation of epithelial sodium channel (ENaC) activity via ROS-induced cysteine modification in the lungs [17]. Other examples include the induction of oxidative stress by numerous environmental toxins due to disruption in cytochrome P450 (CYP) metabolism. Sulfur mustard inhibits NADPH CYP reductase [18]; diesel exhaust particles induce CYP and NADPH quinone oxidoreductase-1 expression, and nuclear factor erythroid 2–related factor 2 (Nrf2) nuclear translocation [19]; and arsenic, asbestos, and tobacco carcinogens elevate CYP expression and activity. These changes affect pulmonary immune/inflammatory responses or contribute to the development of lung cancer [20–22].

### Antioxidant systems

Antioxidants exist as enzymatic or non-enzymatic systems that help restore oxidative balance to maintain cell homeostasis. Superoxide dismutases present in the cytoplasm (SOD1), mitochondria (SOD2), or extracellular compartments (SOD3) catalyze the dismutation of O_2_^•−^ into oxygen (O_2_) and H_2_O_2_. Catalases, present in the cytoplasm and peroxisomes, further catalyze the breakdown of H_2_O_2_ into O_2_ and water, while peroxiredoxins catalyze the reduction of H_2_O_2_. Another class of enzymes in the thioredoxin and glutathione systems includes reductases and peroxidases that detoxify compounds such as ROS and lipid peroxides. These enzymes have been shown to have important protective roles in lung diseases [23–31]. Non-enzymatic antioxidants, present endogenously or by dietary intake, are small molecular weight compounds that scavenge free radicals. Of importance to hypertension, ARDS, asthma, cystic fibrosis, Chronic Obstructive Pulmonary Disease (COPD), infections and cancer are: Glutathione (GSH), a cysteinyl tripeptide; uric acid, an oxypurine produced from xanthine/xanthine oxidase; ascorbic acid (vitamin C), a monosaccharide redox catalyst; and tocopherols/tocotrienols (vitamin E), fat-soluble vitamin that protect membranes from lipid peroxidation radicals [32–38].

#### Physiologic function – oxidants and antioxidants in homeostasis

Endogenous oxidant-antioxidant systems have physiologic functions important in cell homeostasis and cellular adaptation to environmental stress. ROS production as part of the respiratory burst in inflammatory cells has been long recognized to protect against invading organisms; individuals with Chronic Granulomatous Disease are immunocompromised due to defects in the leukocyte to generate O_2_^•−^ via NADPH oxidase (gp91phox or NOX2). The role of NO^•^ as a biologic signaling molecule has also been clearly established, with a role in maintaining vascular tone, neurotransmission and normal immune function. Accumulating new data implicates a key role for ROS in signaling pathways important in multiple processes including proliferation, differentiation, immune function, and vasoregulation. Examples include ROS and 4-hydroxy-2-nonenal (HNE) induced vascular cell proliferation and angiogenesis [39,40], H_2_O_2_ regulation of bone marrow-derived stem and progenitor cell function [41], ROS/RNS regulation of neutrophil and monocyte function [42,43], and ROS signaling that regulate pulmonary vessel tone, kinase-modulated vascular function, and mechanical stretch-induced vascular remodeling [44–46].

### Pathophysiologic function – oxidants/antioxidant imbalance in disease pathogenesis

The pathophysiology of oxidative stress occurs when there is an imbalance in oxidant-antioxidant systems. An accumulation of highly reactive molecules causes generalized damage to DNA, lipids, proteins and carbohydrates. There are well-established methods to measure oxidative stress in disease states, shown for example by increased lipid peroxidation products, DNA oxidation, and protein carbonyl formation in lung tissue. While lipid peroxidation can be a marker of excess ROS production, oxidized lipids are also potent signaling molecules. Isoprostanes, for example, are byproducts of membrane lipid peroxidation that provoke bronchoconstriction and airway hyper-responsiveness in asthma, and powerful vasoconstriction in pulmonary arterial hypertension and acute lung injury [47,48]. Certain highly reactive ROS are associated with indiscriminant oxidative or nitrosative stress, such as hydroxyl radical (^•^HO) or ONOO^−^. In contrast, H_2_O_2_, NO^•^, and O_2_^•−^ have relatively longer half-lives, and specific cellular targets that enable them to function as signaling molecules. Sustained or increased production of these ROS/RNS promotes alterations in cell signaling responsible for disease progression. These species can regulate enzyme function including kinases and phosphatases, G-protein or tyrosine kinase receptors, ion channel function, and transcription factors, resulting in an impact on numerous downstream pathways.

### Overview of redox-regulated signaling

Redox regulated signaling pathways are increasingly recognized as a major mechanism to regulate cellular function. As signaling molecules, ROS and RNS have specific targets that impart their signaling properties and determine their biologic effects. It is well-established that NO^•^ activates guanylate cyclase by binding to the heme moiety, leading to increased cyclic guanosine monophosphate (cGMP)-dependent vasorelaxation. NO^•^ can also lead to vasorelaxation via cGMP-independent mechanisms, for example, by inhibiting the effects of serotonin or alpha-adrenergic agonists on their respective G-protein coupled receptors to blunt vasoconstriction [49]. Both ROS and RNS can directly modify reactive cysteine residues, which represents a major mechanism for redox regulated signaling [50]. Post-translational modifications include disulfide bond formation, reduction, oxidation, nitrosylation, and glutationylation, which alter protein function. Important to lung diseases is S-glutathionylation that uncouples eNOS [51,52] which regulates vascular tone, and S-nitrosylation caused by smoke or chronic airway inflammation in asthma [53,54]. Downstream consequences include modulation a number of cell signal transduction pathways that disturb cell homeostasis [55]. Reactive oxygen or nitrogen species usually have specific targets that are tightly regulated. The reactions are also usually rapid, reversible and occur in specific tissue and cellular compartments. Pathways relevant to lung diseases include regulation of kinase and phosphatase activity on growth factors and growth factor receptors that affect smooth muscle cell proliferation [56,57] or endothelin-1 that mediates pulmonary vasoconstriction [58]; regulation of transcription factors such as nuclear factor kappaB (NFκB), tumor suppressor p53 and hypoxia-inducible factor 1-alpha (HIF-1α) that control expression of genes involved in pulmonary vascular inflammation and remodeling [59–61]; and regulation of molecular adaptors and chaperones such as heat shock protein 90 (HSP90) interactions with eNOS that contribute to endothelial dysfunction associated with pulmonary hypertension [62–64].

NADPH- and GSH-dependent enzymes also play an important role in redox regulated signaling in lung diseases. ROS produced by lipoxygenases and NOX regulate pro-inflammatory responses in allergic airway inflammation [6], while NADPH:quinone oxidoreductase 1 (NQO1) upregulation is a Nrf2-dependent process relevant to macrophage-derived oxidants involved in the pathogenesis of ozone-induced oxidative stress, airway inflammation, and emphysema [65,66]. GSH peroxidases, S-transferase, and reductase modulate GSH and NADP homeostasis which, when altered, induce signaling pathways that promote airway inflammation in COPD and asthma [67–71].

### Strategies to restore redox balance in human disease

**Figure 1:**
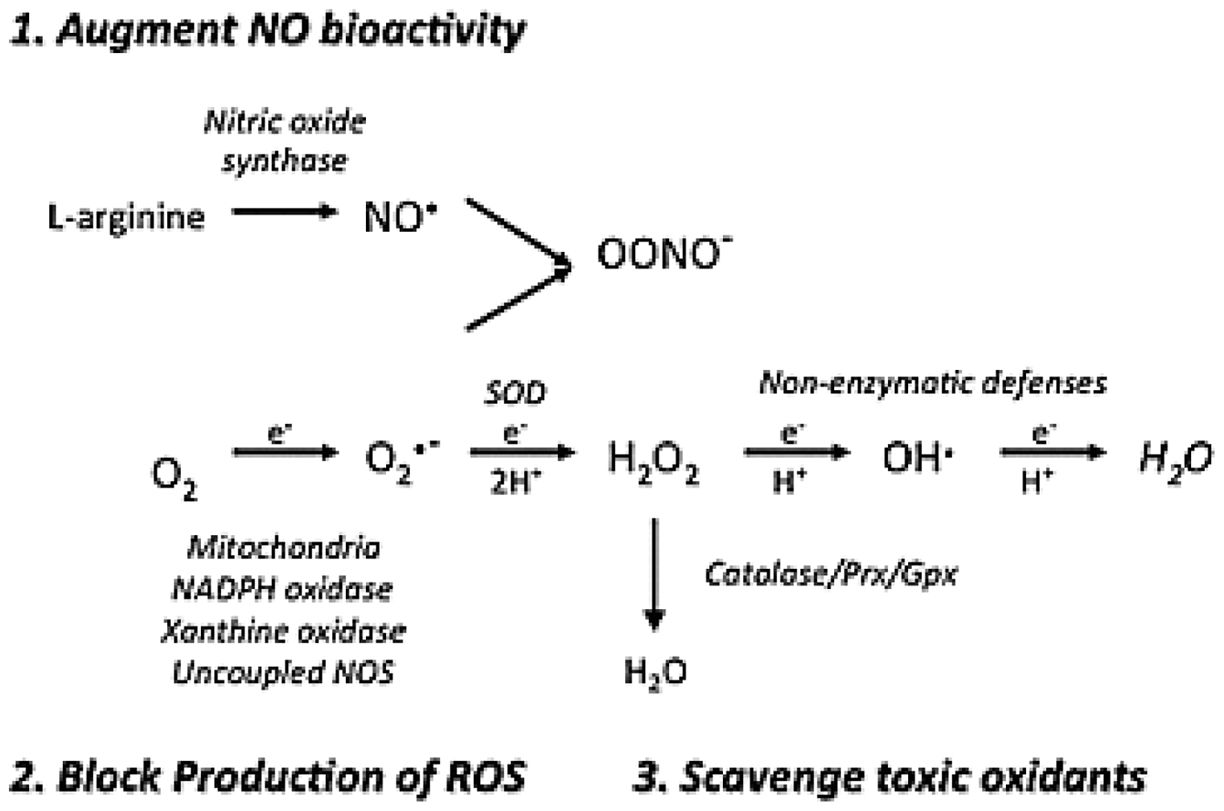
Therapeutic approaches to restore redox balance. 1) Augment NO^•^ bioactivity catalyzed by nitric oxide synthase; 2) Block production of ROS produced by mitochondrial electron transport chain, NADPH oxidase, xanthine oxidase or uncoupled NOS; 3) Scavange toxic oxidants by replacing deficient enzymatic and non-enzymatic antioxidants such as SOD, catalase, GSH, ascorbic acid, tocopherol, and carotenoids.

Multiple clinical trials have tested a range of therapies designed to restore oxidant/antioxidant imbalance. These strategic approaches can broadly be classified as agents that restore NO^•^ bioactivity in the setting of deficient NO^•^; block NO^•^ production in the setting of excess NO^•^; replace deficient antioxidants, in particular GSH and non-enzymatic antioxidants including vitamins and micronutrients; or scavenge ROS (Figure 1). We provide important examples of trials that represent each of these categories of therapeutic approaches, most of which have ultimately had limited or no success in treating lung or pulmonary vascular disease. We propose that there are a number of general problems with the current therapeutic approaches related to the dose and half-life of delivered antioxidants; targeting of the treatment to the proper tissue or cellular compartment; selection of patients based on disease rather than antioxidant status; and disruption of the physiologic role of the oxidants.

### Strategies to augment NO• bioactivity

Based on the role of NO^•^ dysregulation in pulmonary vascular disease and promise in animal studies, a number of therapeutic approaches have been developed to restore NO^•^ homeostasis in the lung and pulmonary circulation including inhaled NO^•^ (iNO), phosphodiesterase inhibitors, and recombinant SOD1. iNO has been studied in pulmonary hypertension as a selective pulmonary vasodilator, in ARDS to improve ventilation-perfusion matching, and in preterm infants to prevent chronic lung disease. While iNO does decrease the need for rescue therapy with extracorporeal life support in full term infants with persistent pulmonary hypertension, it does not improve mortality [72,73]. This remains the only currently FDA approved indication for iNO. iNO failed to improve meaningful clinical outcomes in other clinical settings. iNO treatment for ARDS in adult and pediatric patients showed no change in vent free days or mortality outcomes, and in premature infants, iNO failed to influence later development of bronchopulmonary dysplasia (BPD) [74]. Another strategy is the use of phosphodiesterase 5 (PDE5) inhibitors such as sildenafil, to block breakdown of cGMP, enhancing the activity of the second messenger of NO^•^ responsible for smooth muscle relaxation in airways and vasculature. Sildenafil is an approved therapy for adults with pulmonary arterial hypertension, though its use in pediatric pulmonary hypertension is not recommended due to safety concerns [75–80]. Human recombinant SOD1 has also been tested as a means to increase NO^•^ bioavailability by preventing the inactivation of NO^•^ by O_2_^•−^. In preterm infants, human recombinant SOD was ineffective at improving 28 day mortality infants, though modestly decreased later development of reactive airway disease and possibly decreased retinopathy of prematurity [81–83]. Overall, despite abundant research demonstrating loss of NO^•^ bioactivity in a number of settings, the clinical utility of the current available therapies has been quite limited and may require alternative strategies.

### Strategies to block ROS/RNS production

Though some pulmonary vascular diseases are associated with deficient NO^•^ production, other diseases are characterized by overproduction of ROS or NO^•^, leading to oxidative and nitrosative stress. Numerous laboratory studies of lung and pulmonary vascular disease demonstrate protection when ROS/RNS production is ablated, thus this is another strategy that has also been considered in the clinical research arena. A variety of inhibitors are available that block ROS/RNS production via NOX, xanthine oxidase, NOS, or mitochondria. In the clinical setting, human circulatory shock is characterized by excess production of NO^•^ by inducible NOS, which contributes to catecholamine-refractory hypotension. One multicenter randomized controlled study evaluated a non-specific nitric oxide synthase inhibitor, 546C88 to test its ability to improve hypotension and organ perfusion. Unfortunately, this strategy not only failed to protect, but in fact increased mortality in this patient population [84].

### Strategies to scavenge oxidants

Numerous studies have tackled the problem of oxidative stress by delivering enzymatic or non-enzymatic antioxidant therapies. N-Acetyl cysteine (NAC) is perhaps the most well studied antioxidant, used for over 40 years and possessing multiple antioxidant effects. It acts as a direct powerful free radical scavenger, replenishes depleted GSH stores and also imparts anti-inflammatory effects [85]. Despite these potential beneficial effects, clinical trials using inhaled or intravenous NAC have failed to demonstrate mortality benefit in many diseases such as asthma, ARDS, systemic inflammatory response syndrome or sepsis. However, in some studies, potential improvements in secondary clinical outcomes were observed with NAC, such as faster recovery in ALI [86], improved oxygenation and decreased ventilator [87], and less frequent exacerbations in COPD [88]. In contrast, other studies have raised concerns about cardiac depressant effects of NAC, particularly in patients with sepsis [89]. The utility of NAC in lung injury remains uncertain, with no clear indications for use.

Antioxidant scavenging can be augmented by modifying nutrition, particularly vitamins, trace elements and specific amino acids that have either direct antioxidant effects, serve as precursors or cofactors for antioxidant enzymes, or support immune function. Deficiencies in several antioxidant vitamins including zinc and selenium, and amino acids have been observed in critically ill adult and pediatric patients, and the degree of deficiency often correlates with severity of disease, as is the case with selenium deficiency in severe sepsis [90–93].

Although these dietary factors are promising, readily accessible and easily modifiable targets, results in clinical trials have generally been discouraging. For example, although initial meta-analysis evaluating multiple smaller RCT’s of combination antioxidant micronutrient supplementation suggested an improvement in outcomes, particularly those at high risk of death [94], a subsequent large randomized controlled study showed harm with early glutamine supplementation and no improvement with antioxidants in critically ill patients [95]. Interestingly, in this study the subset of patients randomized to receive selenium were not deficient in selenium, as described in multiple other studies.

### Why have antioxidants failed to cure lung disease?

Despite abundant evidence that oxidative stress is not mere epiphenomena of disease processes, these studies highlight the lack of efficacy with the current antioxidant therapeutic approach in numerous clinical trials. There are a number of reasons why these therapies failed to improve outcomes in human lung diseases. These relate to the selection of the appropriate dose, targeting of the antioxidant to the appropriate tissue or cellular compartment, impact on physiologic function of ROS/RNS, or failing to account for genetic or epigenetic factors or selecting the appropriate patient population. We will review each of these limitations and challenges below.

### Inadequate dose of antioxidants

The ability to deliver the appropriate dose of antioxidant with a suitable half-life poses the first challenge. First, little is known about specific therapeutic levels of antioxidants in which to base dosing regimens. Secondly, due to the need for compensatory increase in antioxidants during times of high oxidative burden, a “therapeutic” level is likely to be a dynamic target depending on the disease state. Guidance for intake of some antioxidant vitamins or nutrients is provided in the form of recommended daily allowances. However, these “allowances” are unlikely to achieve a truly therapeutic level during critical illness, due to higher requirements due to metabolic demands, unpredictable absorption of enteral antioxidants, altered volume of distribution due to capillary leak, and general increased production of ROS/RNS during critical illness. A third challenge in the delivery of antioxidants is the short half-life of endogenous and exogenously supplemented antioxidants, as is the case with recombinant SOD1 [81,83]. This presents a significant challenge in the development of antioxidant enzymatic therapies so that they can be not only safe and efficacious but also appropriately dosed.

### Inadequate tissue delivery

Another consideration in adequately delivering antioxidants is ensuring delivery to the tissue compartment where oxidative stress is occurring. For example, replacing SOD1 intravenously, with a half-life of only a few minutes is unlikely to effectively and adequately restore SOD to the lung tissue [81,83]. In addition, SOD1, due to its negative charge, does not bind to cell surfaces or penetrate tissue well, while SOD3 or the chimeric protein SOD2/3, which are positively-charged, bind to the cell surface and extracellular matrix which improves tissue content and half-life, offering a potential advantage in certain disease settings.

### Inadequate timing of delivery

In addition to delivering a therapeutic antioxidant dose and targeting a specific vulnerable tissue compartment, delivery of antioxidant therapy during a therapeutic window is equally as important. Mechanistically, antioxidants are more likely to be beneficial if started earlier in the course, before the development of irreversible tissue damage occurs.

### Disruption of physiologic function of ROS/RNS

Although antioxidants provide benefit by mitigating damage caused by oxidative stress, interference with the extensive physiologic roles of ROS or RNS by antioxidants may be harmful.

ROS modulate both physiologic and pathophysiologic functions in phagocytosis and immune defense. For example, in a mouse model of systemic inflammatory response syndrome, NOX2 was found to be protective against inflammation, lung injury and mortality [96], while a mouse model of E. coli peritonitis showed increased morbidity and mortality in mice supplemented with vitamin C, GSH and NAC [97]. In contrast, in a mouse model of influenza A pneumonia, NOX2 inhibition resulted in decreased viral titers, decreased airway inflammation, and decreased production of ROS with decreased mortality [98].

ROS/RNS also play a key role in cell growth, accounting for recent evidence that antioxidant therapy can increase cancer risk in both human and animal studies. In the Beta Carotene and Retinal Efficacy Trial (CARET), men and women at high risk for lung cancer who received beta-carotene and vitamin A had a higher incidence of lung cancer versus those receiving placebo [99–102]. In a mouse model of lung cancer, mice supplemented with NAC and vitamin E showed increased tumor progression and decreased survival due to loss of ROS-induced expression of the p53 tumor suppression gene [103]. These examples demonstrate potential detrimental effects on important physiologic processes due to excess scavenging of ROS.

### Lack of consideration of individual factors

The suggestion of potential harm with antioxidants in some patients does not necessarily imply that antioxidant therapy in lung disease should be abandoned, rather that we may need to implement a more individualized approach to the use of antioxidants. Such an approach will require knowledge of individual genetic variations in antioxidant enzymes, epigenetic regulation, and potentially biomarker profiles that identify specific patients vulnerable to oxidative stress and guide patient-specific treatments.

Polymorphisms and genetic variations in numerous antioxidant enzymes have been described. Many of these variations alter antioxidant gene expression, antioxidant protein function or protein distribution, and impact development and progression of respiratory diseases. For example, in premature infants, certain variations in SOD isoforms and catalase are protective against development of neonatal respiratory distress syndrome [104]. In COPD, polymorphisms in antioxidant genes related to GSH function and all isoforms of SOD alter susceptibility to COPD and impact disease progression [105–107]. Genetic variations in antioxidant enzymes have also been implicated in susceptibility to asthma [108] and acute lung injury. Interestingly, a particular polymorphism may have the opposite effect on risk, depending on the disease state. For example, the polymorphisms in SOD3, such as the R213G single nucleotide polymorphism, which shifts the distribution of SOD3 from the tissue to the extracellular fluids, decreases the risk for COPD while increasing the risk for pulmonary vascular disease [109–111]. Knowledge of specific polymorphisms and genetic variations would allow clinicians to target particular vulnerable patients with patient specific antioxidant therapy, rather than large populations with a particular disease. Though, the feasibility of a large study using this more selective approach is difficult, there are small studies that support the notion that those with genetic susceptibility to oxidative stress are more likely to benefit from targeted antioxidant therapy. This was demonstrated by a study of ARDS in which NAC did not offer an overall mortality benefit, however in selected patients with a single nucleotide polymorphism in GSH S-transferase, NAC improved mortality [112].

### New experimental approaches

There are numerous promising approaches currently under investigation that are designed to more effectively restore NO^•^ bioactivity, block excess ROS/RNS production, scavenge ROS/RNS, or address individual variations in antioxidant levels to improve treatment for lung and pulmonary vascular diseases. Many of these therapies are still being tested in the laboratory setting in relevant animal models but will be the foundation for new drug development and study design to treat infants, children and adults with a wide range of lung diseases. This review aims to highlight these general concepts, though is not able to cite the multitude of important investigations in this field.

### Augment NO bioactivity

New approaches to augment NO^•^ bioactivity hold great promise in the treatment of lung and vascular diseases [113]. These approaches include agents that improve delivery or bioavailability of NO^•^, enhance cGMP-dependent NO^•^ signaling, or improve eNOS activity. One of the concerns with NO^•^ delivered as a gas is its high reactivity with O2 in the gaseous phase and with O_2_^•−^ when in the liquid extracellular and intracellular mileau. Delivery of NO^•^ bioactivity through the use of S-nitrosothiols allows for targeted delivery of this important bioactive form of NO^•^. This has widely been done in the laboratory setting using S-nitrosothiols like S-nitrosocysteine, while one potential therapeutic agent is ethyl nitrite, a gas that largely functions as an S-NO donor [55,114–116]. There is also significant interest in the therapeutic use of nitrite to augment NO^•^ bioactivity [117–119]. iNO increases formation of nitrite, nitrate and S-nitrosthiols, while nitrite also is a precursor promoting formation of S-nitrosothiols, which may explain its beneficial effects [120,121]. In addition to new PDE5 inhibitors, guanylate cyclase activators are also under investigation to prolong the biologic activity of NO^•^ [122–126]. Strategies that augment eNOS function to generate NO^•^ include supplementation of substrate or essential co-factors, L-arginine, L-citrulline, or tetrahydrobiopterin (BH(4)) [127–129]. The modulation of BH(4), an essential cofactor in NOS coupling, has also been explored in which BH(4), BH(4) analogs and sepiapterin supplementation was used to increase NO^•^ production and inhibit hypoxia-induced vasoconstriction [130], pulmonary endothelial dysfunction [131,132], and restoring angiogenesis in persistent pulmonary hypertension [133,134]. In addition, inhibition of arginase is another strategy to enhance L-arginine availability for eNOS [135].

### Block ROS/RNS production

Other strategies to selectively block ROS/RNS production by specific enzyme isoforms are being developed experimentally. NOX inhibitors, such as NOX4 inhibitor, have been used to attenuate gene transcripts involved in hypoxia-mediated vascular remodeling and pulmonary fibrosis in rodents [136], and apocynin has been used to inhibit activation of redox transcription factors NFκB and AP-1 and production of pro-inflammatory cytokines TNF-α, IL-1β, and IL-6 in experimental animal models of asthma airway inflammation [137]. Studies have also shown that inhibitors of xanthine oxidase, such as allopurinol, reduce the production of nitrotyrosine in the airways of COPD patients, although exhaled nitric oxide was increased [138]. Potentially a specific NOS2 inhibitor may have benefit in inflammatory states associated with nitrosative states and prevent the issues observed with the general NOS inhibitor described above in the Triumph trial.

### Scavenge oxidants by increasing endogenous antioxidant defenses

A new approach is the induction of endogenous catalytic antioxidants, SOD and catalase, as an antioxidant therapy [139]. This approach has been studied in healthy human subjects that were given a composition of extracts from five medicinal plants (Protandim). Each ingredient has been reported to increase SOD and catalase activity while decreasing plasma TBARS, an indication of decreased lipid peroxidation. The Protandim study evaluated the additive effects of the five-ingredient composition, and showed that after 30 days of supplementation TBARS was decreased by 40%, and after 120 days erythrocyte SOD increased by 30% and catalase increased by 54%. Protandim functioned by increasing endogenous Nrf2 antioxidant defenses. Nuclear factor (erythroid-derived 2)-like 2, Nrf2, is a master regulator of the human Antioxidant Response Element (ARE), serving as a transcription factor for the genes of a number of antioxidant enzymes. In normal conditions, Nrf2 resides in the cytoplasm bound to Kelch like-ECH-associated protein 1 (Keap1) and Cullin3, and is ultimately ubiquinated and degraded. As a stress response, cysteine residues in Keap1 disrupted, causing Nrf2 to be released and translocate into the nucleus to bind to ARE. When Nrf2 is activated, antioxidant-related genes involved in several lung related diseases such as lung inflammation, pulmonary fibrosis, pulmonary hypertension, acute mountain sickness, and lung cancer are expressed. In addition to Protandim, other approved therapeutic agents may also increase Nrf2 activation [140–143].

### Targeted therapies

An increased understanding of the pathophysiology of lung diseases related to oxidative stress has lead to the development of therapies that have potential to be more effective and efficient by targeting specific lung compartments and cell types. Administration of therapeutics by inhalation for localized effects in the lung has long been a conventional method. Related to direct lung delivery of antioxidants, both aerosolized recombinant SOD3 and a novel SOD2/3 chimeric protein delivered intratracheally in rodents showed protection from hyperoxia or acute hypoxia [144,145]. Recent progress in the development of inhalable delivery systems include micro- and nanoparticles that show increased, stable, or sustained release of encapsulated drug in the lung [146–148], which provide the promise of applying these technologies to antioxidant delivery to the lung [149]. Furthermore, advancements in pharmaceutical biotechnology has allowed the development of other lung targeted delivery systems [150] that can be administered systemically, and novel antioxidant therapies with improved targeting capacities in the lung. Antibody conjugated proteins and nanoparticles that target ICAM-1 or PECAM-1 receptors on pulmonary endothelium has been used to deliver NOX inhibitors, SOD and catalase to protect against oxidative stress in the pulmonary vasculature [151–154]. Other modern drug delivery strategies utilize redox-responsive carriers to target and release drug within redox microenvironments [155].

## Harnessing Personalized Medicine

As discussed above, many studies of antioxidant therapies selected patients broad groups of patients who were all vulnerable to oxidative stress from lung disease, but it is plausible that a more personalized and targeted approach to antioxidant therapy using known genetic variations in antioxidants, known epigenetic changes and perhaps particular biomarker profiles would better target patient-specific therapies to improve outcomes. Knowledge of polymorphisms and genetic variations that affect antioxidant expression, function, and tissue distribution may allow targeted therapy to the appropriate individuals to replete deficient antioxidants.

Another approach to better target antioxidant therapies is to utilize available biomarker profiles to tailor specific therapy. There are multiple measurable markers of both oxidative stress and antioxidant enzyme activity. There is clearly no benefit in augmenting antioxidant defenses if they are not deficient, and there may in fact be harm, as discussed above. Although there are challenges with this approach, knowledge of particular antioxidants or particular markers of oxidative stress will likely prove to be clinically relevant and guide therapy. The application of exhaled nitric oxide (eNO) measurements provides an example of how this approach may be useful. Noninvasive measurements of eNO reflect derangements in NO^•^ and inflammation [156]. In asthma, where eNO has been most well studied, elevations in eNO correlate with degree of airway inflammation and bronchial hyperreactivity, and helps guide use of asthma controller medications [157]. In sickle cell disease, eNO inversely correlates with the degree of severe airway obstruction and pulmonary hypertension [158], as well as inflammatory pulmonary diseases including Cystic Fibrosis (CF) and non-CF related bronchiectasis, bronchopulmonary dysplasia, and bronchiolitis [159–161]. Other biomarkers of oxidative stress can be assessed through exhaled breath condensates and this is an area of active research that may guide other antioxidant therapies [161].

In conclusion, an imbalance between production of ROS/RNS and scavenging capabilities through enzymatic and non-enzymatic defenses is implicated in diverse lung and pulmonary vascular diseases. The therapeutic approach to treat oxidative stress has encountered major barriers that we propose are complicated by the inadequate delivery of the proper antioxidant in the right concentration to the appropriate tissue or cell compartment. It is now clear that since ROS/RNS are critical biologic signaling molecules essential to cell homeostasis and adaptation to stress, indiscriminant scavenging of these molecules may decrease ROS levels but actually worsen the disease process by disrupting normal cellular functions. Furthermore, as personalized medicine evolves, it will be essential to consider individual genetic or epigenetic factors impacting the oxidant/ antioxidant system to more appropriately guide therapy. Novel therapeutic agents bring exciting opportunities to harness new knowledge and utilize targeted and patient specific therapies in the future to treat lung and pulmonary vascular diseases.
